# Management of Brain Metastases in Epidermal Growth Factor Receptor Mutant Non-Small-Cell Lung Cancer

**DOI:** 10.3389/fonc.2018.00208

**Published:** 2018-07-03

**Authors:** William J. Kelly, Neil J. Shah, Deepa S. Subramaniam

**Affiliations:** Division of Hematology-Oncology, Georgetown University, Washington, DC, United States

**Keywords:** epidermal growth factor receptor, non-small-cell lung cancer, brain metastases, targeted therapy, osimertinib

## Abstract

Lung cancer remains a leading cause of mortality with 1.69 million deaths worldwide. Activating mutations in epidermal growth factor receptor (EGFR), predominantly exon 19 deletions and exon 21 L858R mutations, are known oncogenic drivers identified in 20–40% of non-small-cell lung cancers (NSCLC). 70% of EGFR-mutant NSCLC patients develop brain metastases (BM), compared to 38% in EGFR wild-type patients. First-generation tyrosine kinase inhibitors (TKIs), such as erlotinib and gefitinib have proven to be superior to chemotherapy in the front-line treatment of EGFR-mutant NSCLC, as has afatinib, a second-generation TKI. The most common acquired resistance mechanism is the development of a gatekeeper mutation in exon 20 T790M. Osimertinib has emerged as a third-generation EGFR TKI with proven activity in the front-line setting as well as in patients with a T790M acquired resistance mutation with remarkable CNS activity. As long-term survival outcomes in EGFR-mutant NSCLC continue to improve, the burden of BM becomes a greater challenge. Here, we review the literature related to the management of BM in EGFR-mutant NSCLC including the role of the three generations of EGFR TKIs, immunotherapy, and brain radiation.

## Overview of Brain Metastases in Epidermal Growth Factor Receptor (EGFR) Mutant Non-Small-Cell Lung Cancer (NSCLC)

### Epidemiology and Molecular Alterations in EGFR Mutant NSCLC

Lung cancer remains a leading cause of mortality with 1.69 million deaths worldwide (1). An estimated 234,030 new cases will occur in the United States in 2018 with a median age at diagnosis of 70 and 64% predominance for males (2). Approximately 84% of these lung cancers are non-small cell lung cancers (NSCLC) (3). NSCLC has traditionally been classified by histology (adenocarcinoma, squamous, and large cell) but the classification paradigm has evolved to incorporate molecular subtypes that guide treatment decision making.

Epidermal growth factor receptor (EGFR) is a transmembrane tyrosine kinase receptor which activates Jak, PI3K, ROS, and RAS pathways leading to cell survival (4, 5). The most common activating mutations are exon 19 deletions or point mutations in exon 21 *via* Leu858Arg (L858R) (6, 7). Reports of the prevalence of EGFR mutations in NSCLC ranges from 46.7% in the East Asian population as reported by Liu et al. (8) to 38.4% (range 36.5–40.3%) in China and 14.1% (range 12.7–15.5%) in Europe seen in Zhang et al. (9) and 22% in African Americans enrolled in the Lung Cancer Mutation Consortium (10). The landmark BR21 trial demonstrated the survival advantage in chemo-refractory NSCLC with the use erlotinib, a first-generation EGFR inhibitor (11). Subsequently, three additional drugs (gefitinib, afatinib, and osimertinib) have now been approved to treat newly diagnosed EGFR-mutated advanced NSCLC. Among NSCLC patients who progress on first- or second-generation EGFR TKI therapy, most do so through a unique gatekeeper mutation, viz. the exon 20 point mutation Thr790Met (T790M) in the ATP-binding site of EGFR (12). Incidence of the T790 gatekeeper mutation has been reported to be between 49 and 63% (13, 14). The methionine side chain acts as a “gatekeeper” residue causing steric hindrance thus decreasing hydrophilicity and preventing tyrosine kinase binding (15). The T790M mutation also increases ATP affinity (16). Other rare mechanisms of TKI resistance include MET amplifications or mutations, HER2 amplifications, and rarely BRAF mutations (12). Additionally, transformation to small cell histology is another possible mechanism of EGFR TKI resistance (13).

### Prevalence of Brain Metastases (BM) in EGFR-Mutant NSCLC

Among NSCLC patients, those with BM have an increased frequency of EGFR mutations than those without brain metastasis and conversely, among EGFR mutant NSCLC patients the incidence of BM (70%) greatly surpasses the incidence of BM in wild-type (wt) EGFR NSCLC patients (38%) (17). Approximately, one-third of EGFR-mutant NSCLC patients develop central nervous system (CNS) progression during the course of their illness (18). Among Asian populations, the prevalence of EGFR mutations in NSCLC BM ranges from 39 to 63% (19, 20). Among North American and European populations this ranges from 2 to 40% (21, 22). At initial diagnosis, EGFR mutation discordance estimates between primary and BM range are minimal (23). Prevalence of T790M mutations in CNS lesions among EGFR mutant NSCLC patients with TKI failure is much lower than anticipated at around 17% (24). This may reflect a pharmacokinetic failure of the first-generation EGFR TKIs to penetrate the brain and thus induced acquire resistance *via* the gatekeeper T790M mutation. Case reports have detailed patients on gefitinib and erlotinib, first-generation TKIs with modest brain penetrance, who have developed T790M-mediated resistance at primary tumor locations but not in the brain metastasis (25, 26). CNS progression appears to be higher in those with L858R point mutations (18). Interestingly, a retrospective radiologic analysis of 57 NSCLC patients suggested that exon 19 deleted patients may have more of a miliary pattern of BM (27). Table 1 summarizes the prospective trials of three generations of EGFR tyrosine inhibitors in EGFR-mutant NSCLC with BM.

**Table 1:** Prospective studies in epidermal growth factor receptor (EGFR) mutant non-small-cell lung cancers (NSCLC) patients with brain metastases (BM).

Study	Phase	Tyrosine kinase inhibitors therapy	EGFR mutant NSCLC patients with BM (unless specified)	Response rate (%)	Survival (months)
Park 2012	II	Erlotinib or gefitinib	28	Partial Response (PR): 83 Stable Disease (SD): 11	Progression-free survival (PFS): 6.6 Overall Survival (OS): 15.9

Yu 2017	I	Pulsatile erlotinib	34 (only 32% had brain mets)	Complete Response (CR): 2 PR: 70	PFS: 9.9

Iuchi 2013	II	Gefitinib	41	Objective response rate (ORR): 88	PFS: 14.5 OS: 21.9

Yang 2017 (BRAIN)	III	Icotinib	85	–	Intracranial PFS: 10.0

Schuler 2016 (LUX-Lung 3/6)	III	Afatinib	25/46	–	PFS: 11.1/8.2

Park 2016 (LUX-Lung 7)	II	Afatinib	26	–	8.4

Mok 2017 (AURA 3)	II	Osimertinib	144 (T790M mut)	–	PFS: 8.5

Goss 2017 (AURA/AURA2)	II	Osimertinib	50 (T790M mut)	Central nervous system (CNS) ORR: 54	–

Yang 2017 (BLOOM)	I	Osimertinib	32 (LM, 11 T790M mut)	ORR: 43	–

Soria 2017 (FLAURA)	III	Osimertinib	53	ORR: 75 CNS PD: 6	PFS: 15.2

## First-Generation Tyrosine Kinase Inhibitors (TKIs)

### Erlotinib

Erlotinib is a first-generation (EGFR) tyrosine kinase inhibitor (TKI) (28). The drug reduces EGFR autophosphorylation in intact tumor cells at a median inhibitory concentration of 20 nM although this ranges from 5 (nM) and 6 (nM) in exon 19 deletion and L858R cell models respectively to >2,000 (nM) in T790M models (29, 30). High-performance liquid chromatography studies have shown that erlotinib penetrates the cerebrospinal fluid (CSF) at a rate of between 2 (67 nM) and 4% (31, 32). Radiolabeled ^11^C-erlotinib injected to one NSCLC patient was shown to accumulate in brain metastasis (33). Additionally, the average concentrations of erlotinib in CSF appear to be higher in those with partial responses (PRs) (35 ng/ml) compared to those who have progressive disease (16 ng/ml) (32).

Several studies have evaluated the effect of erlotinib in NSCLC patients with BM. Deng et al. reported on six unselected NSCLC patients with BM treated with erlotinib and noted that four of the six harbored an EGFR mutation in the tumor; two PRs and two stable diseases (SD) were noted in EGFR mutant patients (32). Porta et al. retrospectively reviewed 69 NSCLC with BM patients treated with erlotinib (34). 17 patients had EGFR mutations, 82% of whom had an objective response rate (ORR) to erlotinib including eight complete responses (CRs) as well as a median time to progression of 11.7 months compared to 5.8 in EGFR wt patients and an overall survival of 12.9 months versus 3.1, respectively (34). Moreover, no patients without EGFR mutations had an objective response (34). A phase II study prospectively evaluated EGFR mutant NSCLC patients treated with erlotinib or gefitinib and noted that 83% achieved a PR and 11% SD without a statistically significant difference in progression-free survival (PFS) (6.6 months) or overall survival (OS) (15.9) between the two TKIs (35).

Dose escalation has also been examined as a potential strategy to increase CNS permeability and overcome resistance. In a small but compelling retrospective case series of nine EGFR mutant lung cancer patients with brain or leptomeningeal metastases that occurred despite conventional dosing of an EGFR inhibitor, patients were treated with high dose “pulsatile” erlotinib (1,500 mg weekly) and a CNS partial response rate of 67% (6 of 9 patients) was noted; however, median time to CNS progression was only 2.7 months (36). Following this, a phase 1 study in 34 patients with EGFR mutant lung cancer treated with escalating pulse doses of erlotinib found the maximum tolerated dose to be 1,200 mg given on days 1 and 2, with 50 mg given on days 3–7 weekly and it should be noted that 32% of patients had BM at study entry and none of these patients had progression of an untreated CNS metastasis or new CNS lesions while on study (37).

The role of erlotinib in leptomeningeal disease has also been examined. A retrospective review of 25 NSCLC (9 with exon 21 EGFR mutation and 8 with exon 19 deletion) patients with leptomeningeal (LM) carcinomatosis treated with either erlotinib or gefitinib demonstrated that those treated with erlotinib had a cytologic conversion rate of 64.3% compared to 9.1% with gefitinib (38), suggesting greater activity of erlotinib over gefitnib in the setting of LM disease. In another series of NSCLC patients with leptomeningeal metastasis who had failed gefitinib treatment, all 6 patients with an EGFR mutation-derived clinical benefit with 3 PRs and 3 with SD (39). 1 patient whose tumor did not harbor an EGFR mutation developed progressive disease as the best response.

### Gefitinib

Gefitinib, another first-generation EGFR TKI, is a substrate for the *P*-glycoprotein efflux pumps and the drug has a brain penetration rate of only 1% (40, 41). There have been many retrospective reviews of NSCLC patients with BM treated with gefitinib. An old retrospective study of 14 NSCLC patients with BM observed 1 CR (6%) and 5 PRs (33%); this was done prior to the understanding of the role of EGFR mutation status on response to targeted therapies (42). Another report on 15 patients found an ORR of 60% (43). In 2009, a retrospective study of 23 Korean never-smoker patients with lung adenocarcinoma and brain metastasis without prior whole brain radiotherapy (WBRT) found that gefitinib or erlotinib without WBRT resulted in an intracranial response rate of 73.9%, noting that the prevalence of EGFR mutations in Korean non-smoker NSCLC population is high (44). Following this, Zhang et al. retrospectively reviewed 43 Chinese EGFR mutant NSCLC patients with BM treated with gefitinib or erlotinib until extracranial lesion progression; an intracranial lesion ORR of 57% and PFS of 9.3 months was observed, with no statistically significant difference in OS between gefitinib versus erlotinib (45).

Multiple prospective studies have also shown efficacy of gefitinib in NSCLC patients with BM. In 2004, Ceresoli reported on 41 NSCLC patients with BM treated with gefitinib including 18 patients with prior WBRT and observed a partial response rate of only 10% (46). Chiu et al. conducted a prospective study in 57 unselected NSCLC with BM patients observing an ORR of 33% and PFS of 5 months (47). Similarly, a 2007 study by Wu et al. examined 40 unselected NSCLC with BM patients (23 with prior WBRT) and found a 32% ORR and PFS of 9 months (48). However, a phase II study in 21 Chinese NSCLC patients with BM treated with prior WBRT reported a much higher 81% ORR and a PFS of 10 months (49).

Subsequent studies focused on gefitinib’s efficacy in NSCLC with BM patients who harbored EGFR-activating mutations. Iuchi reported in 2013 on a phase II trial of 41 Japanese lung adenocarcinoma patients with BM showing a brain metastasis ORR of 87.8% with 13 CRs (50). Stereotactic radiation and WBRT were required in 20 patients (50). Patients with exon 19 deletions had a statistically significant PFS and OS advantage compared to L858R mutations (50). While the results of many of these studies appeared to be promising, the results and thus, the potential efficacy of first-generation EGFR TKIs in patient with BM, need to be interpreted with caution given the small numbers of patients in these predominantly retrospective reports.

### Icotinib

Icotinib, a first-generation TKI approved in China, has a median CSF penetration rate of 6.1% (51). The BRAIN study was a multicenter, open-label, parallel randomized controlled trial of 176 Asian EGFR mutant NSCLC patients with at least three brain lesions; patients treated with icotinib had a median intracranial PFS of 10.0 months compared to 4.8 months in those treated with whole brain irradiation plus concurrent or sequential chemotherapy, translating to a 44% risk reduction from intracranial progression or death, and making this a potentially promising option (52).

## Second-Generation TKIs

### Afatinib

Afatinib is an oral second-generation TKI which selectively and irreversibly blocks EGFR, HER2, and HER4 kinase activity (53–55). The LUX-Lung 3 was a phase III trial of front-line afatinib in EGFR mutant advanced or metastatic NSCLC (56). Subgroup analysis of 35 patients with asymptomatic BM showed a PFS of 11.1 months versus 5.4 months with cisplatin and pemetrexed (57). The LUX-Lung 6 study was an open label randomized, multicenter phase III trial of Asian patients with EGFR mutant advanced or metastatic lung cancer (58). Prespecified subgroup analysis of 46 asymptomatic BM patients revealed that PFS was improved from 8.2 to 4.7 months in those treated with gemcitabine and cisplatin (58). Those who received whole brain radiation therapy appeared to have better PFS benefit than those who did not receive radiation; however, among BM patients, rates of CNS progression were similar to chemotherapy in both the LUX-Lung 3 (45 vs 33% chemotherapy) and LUX-Lung 6 (21.4 vs 27.8%) (58).

Another study through the afatinib compassionate use program examined 100 NSCLC patients with BM and/or leptomeningeal disease who had progressed on platinum chemotherapy and a first-generation EGFR TKI, 74% of whom had a documented EGFR mutation (59). Median time to treatment failure was 3.6 months and was similar to a matched group of 100 patients without CNS metastasis; 35% had cerebral response and one heavily pretreated patient with impressive leptomeningeal response and neurological recovery had a CSF concentration of nearly 1 nM (59).

The LUX-Lung 7 study, which was an international, open label, randomized phase II trial comparing afatinib to gefitinib in EGFR mutant advanced or metastatic NSCLC patients, noted that PFS was longer with afatinib (11 months) than gefitinib (10.9 months) but not statistically significant among the subgroup of 26 patients (16%) with asymptomatic brain metastasis (60). Thus, despite its promise as a second-generation irreversible EGFR targeted agent, afatinib did not pan out to be significantly superior to the first-generation agents in systemic disease (or CNS disease) and its use is limited by its greater toxicity profile, except in some of the less common EGFR mutations where data suggest better efficacy.

### Dacomitinib

Dacomitinib, another second-generation TKI with activity against EFGR, HER2, and HER4 was studied in two double blind, multicenter, randomized phase III trials: BR.26 and ARCHER 1009 (61, 62). In BR.26, dacomitinib did not improve OS in patients who had previously received gefitinib or erlotinib; routine brain imaging was not done in this study (61). Similarly, in Archer 1009, dacomitinib was not superior to erlotinib in advanced or metastatic NSCLC and only 2% of patients in this trial had brain metastasis at baseline (62).

## Third-Generation TKIs

### Osimertinib

Osimertinib is an oral, irreversible EGFR TKI that targets the classical activating mutations as well as the gatekeeper resistance mutation, i.e., T790M (63). Preclinical models showed that osimertinib had greater penetration of the murine blood–brain barrier (BBB) than gefitinib, rociletinib, or afatinib and increased exposure by labeled radiography in cynomolgus monkey brains (64).

AURA 3 was a randomized, international, open-label, phase II trial of T790M positive advanced NSCLC patients who had progressed on front-line EGFR TKI therapy (63). 144 patients had CNS metastases and those who received osimertinib had a longer median PFS (8.5 months) than platinum-pemetrexed chemotherapy (4.2 months) with a hazard ratio of 0.32 (63). A pooled analysis of AURA extension and AURA2 trials in 50 patients with asymptomatic BM found a CNS ORR of 54% to osimertinib treatment with 12% having complete CNS response, with benefit also noted in patients who had not received prior radiotherapy to the brain (65).

The BLOOM study was a phase I trial of patients with CSF cytology confirmed leptomeningeal disease (66). Preliminary results of 32 treated patients (23 evaluable) found 10 had radiographic improvement and 13 with SD; additionally 7 of 8 symptomatic patients improved and, the geometric mean decrease in CSF EGFRm DNA copy was 57% (66).

The FLAURA study is a phase III study in 556 EGFR mutant (exon 19 del or L858R) advanced NSCLC patients which randomized patients 1:1 to a standard of care EGFR TKI (erlotinib or gefitinib) or osimertinib (67). Patients with neurologically stable CNS metastases were allowed on this study, accounting for 21% of patients on this study (67). Front-line treatment with osimertinib resulted in improved median PFS (18.9 months) compared to standard EGFR TKI therapy with erlotinib or gefitinib (10.2 months); median OS data were not mature at the time of the PFS analysis (67). It should be noted that ORR of known/treated CNS metastasis at trial entry was 77% in the osimertinib-treated patients compared to 63% in the standard EGFR TKI patients (68). Response duration lasting >or = 6 months was noted in 88% of patients on the osimertinib arm, with a CR rate of 18%, while no CRs were observed in the arm with standard EGFR TKI. This has led to the FDA approval of osimertinib in the United States as an option for the front-line treatment of EGFR-mutated NSCLC harboring exon 19 deletions or exon 21 L858R mutations.

### Rociletinib

Rociletinib (CO1686) was a unique, oral, irreversible TKI designed for NSCLC patients with activity against activating EGFR mutations (L858R and Del 19) and the gatekeeper resistance mutation T790M. The CNS activity of rociletinib was poor compared to systemic disease (69, 70). Camidge et al. reported that 22 of 42 patients continued rociletinib for an average of 120 days after CNS disease progression, treated with brain radiation (70). The development of this drug has since been halted given the high risk-to-benefit ratio related to the hyperglycemia resulting from blockade of the insulin growth factor receptor. Importantly, rociletinib appears to have poor brain penetration with most patients on rociletinib coming off the drug for CNS progression. In a study of the clinical activity of osimertinib in 45 EGFR-mutant NSCLC patients previously treated with rociletinib, subsequent treatment with osimertinib still achieved a brain disease control rate (response + SD) of 88% (71).

## Miscellaneous TKIs

### AZD3759

AZD3759 in an oral EGFR TKI designed for CNS penetration with a ratio of unbound brain to unbound plasma concentration of 0.65 (72, 73). AZD3759 caused tumor regression in leptomeningeal and brain metastasis mouse models (73). Preliminary results of the phase I BLOOM study of 38 EGFR-mutant NSCLC with BM or leptomeningeal metastasis (LM) treated with AZD3759 showed an intracranial ORR of 63% and extracranial ORR of 50% (74). Trough CSF concentrations were above the IC90 for pEGFR (74). Further development of this drug is on hold given the highly promising results with osimertinib in the FLAURA trial.

### Tesevatinib

Tesevatinib (KD019) is a novel, oral, reversible TKI, which inhibits EGFR, HER2/neu, and Src family nonreceptor tyrosine kinases. Preclinical studies demonstrated good blood–brain penetration of tesevatinib with brain/blood radioactivity of 1 at 6–24 h and brain/plasma ratio of 2.3–4.4 from 1 to 24 h after a dose of tesevatinib (75). The studies also reported good anti-tumor activity with extended median survival time by 20% in preclinical mice models. Considering the preclinical results, Berz D et al. enrolled NSCLC pts with EGFR activating mutation and BM (*n* = 4) or leptomeningeal metastases (LM) (*n* = 3) which had progressed after prior EGFR TKI therapy (76). The authors used RECIST 1.1 for BM measurement and response evaluation. Symptomatic LM disease was diagnosed with CSF cytology or MRI finding and response was measured by improvement in symptoms, CSF cytology, and/or MRI findings. One patient with BM had 19% reduction of target lesion on day 23, and another patient had 57% reduction of target BM with resolution of LM symptoms and MRI findings on day 41. Grade ≥ 3 AEs were QTc prolongation, hypokalemia, dehydration, UTI, and ALT elevation.

## Combinatorial/Alternative Treatment Approaches

### Combination EGFR TKI and Radiotherapy

Brain metastasis are resistant to systemic chemotherapy due to the BBB which restricts passage of small, non-polar molecules, or those with receptor-mediated transport (77). Thus research has investigated whether radiation can enhance TKI efficacy. In preclinical models, EGFR TKIs have been shown to increase radiation responses by promoting radiation-induced apoptosis as well as inhibiting cellular cycling, DNA damage repair, accelerated repopulation, and angiogenesis (78–80).

Concurrent use of EGFR TKIs during radiotherapy remains in question. A retrospective study of 44 EGFR-mutant NSCLC treated with concurrent radiotherapy evaluated adverse events (AEs) (81). The most common AEs were rash (50%), anorexia (18%), and diarrhea (15%) with two patients having grade ≥ 3 rash (81). Radiation-related AEs included hydrocephalus (2 patients), pneumonitis (3 patients, one grade ≥ 3), myocarditis (1 patient), radiodermatitis (3 patients), laryngopharyngitis (2 patients), esophagitis (2 patients), and enteritis (1 patient) (81). A meta-analysis of 9 retrospective studies and 1 randomized controlled trial examining WBRT with EGFR TKI versus WBRT alone or EGFR TKI therapy alone included 1,041 unselected NSCLC patients with BM (82). In comparing combination therapy versus EGFR TKI alone the hazard ratios showed improved intracranial PFS with EGFR TKI alone (82). In comparing combination therapy versus WBRT alone the combination therapy had significantly improved OS (HR 0.52), intracranial PFS (HR 0.36), and extracranial PFS (HR0.52) (82). In addition, another meta-analysis of 15 studies including 3 phase II and 1 phase II trials in 1,552 unselected NSCLC patients with BM found that combination radiotherapy and EGFR TKI had improved response rate and disease control rate than radiotherapy alone or chemotherapy (83). Combination therapy significantly prolonged time to CNS progression (HR 0.56) and median OS (HR 0.58) but increased AE including rash (83).

A 2015 meta-analysis examined 12 observational studies that exclusively included EGFR-mutant NSCLC patients with brain metastasis (84). The analysis found that upfront cranial radiation improved intracranial PFS and 2-year OS but more neurological AEs were noted (84). One retrospective review by Gerber et al. examined 222 EGFR-mutant NSCLC BM patients treated with erlotinib, or WBRT or stereotactic radiation (SRS) (85). Patients treated with SRS had an OS of 64 months which was statistically significantly longer than the erlotinib group with median OS of 26 months (85). The results are likely biased due to the selection of patients with lower intracranial disease burden for the SRS approach. The median time to intracranial progression was understandably longer in the WBRT arm than the upfront erlotinib arm (24 vs 16 months; *p* = 0.04) (85). Another multi-institutional analysis of 351 EGFR-mutant NSCLC patients with BM compared treatment with SRS followed by EGFR TKI, WBRT followed by EGFR TKI, or EGFR TKI followed by radiotherapy (SRS or WBRT) at intracranial progression (86). Those receiving SRS upfront had improved OS (46 months) compared to those receiving upfront WBRT followed by TKI (30 months), or upfront EGFR TKI (25 months) (86).

### Immunotherapy

PD-1 blockade has revolutionized the treatment of lung cancer and has been shown to have intracranial responses. However, many of the landmark immunotherapy studies have excluded EGFR-mutant NSCLC patients or patients with BM. Early analysis from a non-randomized, open-label, phase II trial showed 33% brain metastasis response rate among 18 NSCLC with BM (87). However, only one patient in this study had EGFR mutation (87). *In vitro* studies have shown that PD-L1 protein expression is higher in EGFR-mutant NSCLC cell lines than in EGFR wt and expression of mutated EGFR can induce PD-L1 expression (88, 89). In NSCLC, estimates of brain metastasis PDL1 positivity (PDL1 tumor cell expression exceeding 5%) have ranged from 12 to 52% (90–92) but this has not been well characterized in the EGFR-mutant population.

Given the potential for intracranial activity the question may arise if checkpoint inhibition has a role in EGFR-mutant NSCLC with BM. While there is a paucity of data for checkpoint inhibitors in this population, some extrapolation from EGFR-mutant NSCLC is possible. A meta-analysis of Checkmate 057 (nivolumab), Keynote 010 (pembrolizumab), and POPLAR (atezolizumab) showed that immune checkpoint inhibition prolonged OS over docetaxel in EGFR wt but not EGFR-mutant NSCLC (93). As checkpoint inhibition does not appear superior to chemotherapy EGFR-mutant NSCLC, immunotherapies use in the EGFR-mutant NSCLC with BM population is likely equally reserved. Nevertheless, one should consider immunotherapy in later lines of therapy.

### Combinational EGFR TKI and Anti-Angiogenic Therapy

Several studies have looked at combining EGFR TKI with vascular endothelial growth factor directed monoclonal antibody therapy (94). The BELIEF trial was an international, multicenter, single-arm phase II trial of 109 treatment-naïve, advanced or metastatic, EGFR-mutant, lung adenocarcinoma patients treated with the combination erlotinib and bevacizumab (95). 37 patients (33%) harbored T790M mutations and 21 (19%) had brain metastasis; the median PFS was 13.2 months overall and 8.8 months for patients with brain metastasis (95). One of the greatest concerns with bevacizumab use among brain metastatic patients has been CNS hemorrhage. While CNS hemorrhage carries high morbidity and mortality, the incidence of CNS hemorrhage among bevacizumab-treated patients is less than 0.2% (96). Ongoing studies are investigating the combination of osimertinib and bevacizumab in EGFR-mutant NSCLC with BM (NCT02971501).

## Conclusion

In conclusion, patients with EGFR-mutant NSCLC are continuing to live longer with median overall survival of 30.9 months and nearly 15% of patients are alive at 5 years (97). As patients live longer, most of these patients are likely to develop BM and we will need optimal therapies with low toxicity to manage the BM. Based on the summary of literature to date (Table 1), it is the expert opinion of the authors that a CNS-active TKI such as osimertinib is the EGFR TKI of choice in newly diagnosed advanced NSLC harboring exon 19 deletions or exon 21 L858R mutations, given not only its CNS response rate but the durability of the CNS control, in addition to compelling data with tripling of the median PFS. It would be hard to argue against our opinion that osimertinib is the drug of choice in patients with and without BM. That said, the data regarding the use of upfront SRS followed by EGFR TKI needs to be taken into account in personalizing treatment options for patients with EGFR-mutant NSCLC and BM. The EGFR-mutant NSCLC patient who presents with a solitary brain metastasis should still be considered for surgical resection followed by CNS-active EGFR TKI therapy such as osimertinib. Selected patients with CNS oligometastatic disease with large volume BM that are symptomatic may benefit from the Magnuson approach of using upfront SRS while those with military or multiple, small, and especially asymptomatic BM may be able to delay the need for radiation with the use of upfront EGFR TKIs such as osimertinib. Whole brain radiation should be an option that is reserved for refractory BM that have progressed beyond SRS and systemic therapies, thus delaying the onset of neurocognitive decline that almost inevitably follows such an approach. Novel WBRT techniques such as hippocampal sparing (RTOG 0933) or use of drugs such as memantine (RTOG 0614) may further help to reduce the long-term neurotoxicity of WBRT in these patients (98).

## Author Contributions

WK drafted epidemiology, prevalence, TKI, radiotherapy, immunotherapy, and anti-angiogenic therapy sections. NS provided literature review and drafted TKI sections. DS provided concept, design for paper, scientific review, and interpretation of data. All authors contributed to manuscript revision, read, and approved the submitted version.

## Conflict of Interest Statement

WK and NS do not have personal, professional, or financial relationships that constitute conflict of interest. DS has received honoraria for her role as advisory board member or speaker for Bristol Myers-Squibb, Takeda Oncology, and Astra Zeneca.
